# No-Touch Saphenous Vein as Y-Composite vs Aortocoronary Graft: Early Outcome Analysis

**DOI:** 10.1016/j.atssr.2023.09.015

**Published:** 2023-10-04

**Authors:** Suk Ho Sohn, Yoonjin Kang, Ji Seong Kim, Jeehoon Kang, Ho Young Hwang

**Affiliations:** 1 Department of Thoracic and Cardiovascular Surgery, Seoul National University Hospital, Seoul National University College of Medicine, Seoul, Republic of Korea; 2 Department of Internal Medicine and Cardiovascular Center, Seoul National University Hospital, Seoul National University College of Medicine, Seoul, Republic of Korea

## Abstract

**Background:**

A randomized controlled trial was designed to compare 1-year morphologic changes of the no-touch saphenous vein (SV) as a Y-composite graft (composite group) vs an aortocoronary graft (aorta group) in coronary artery bypass grafting. This study evaluated early clinical and angiographic outcomes as a preliminary analysis.

**Methods:**

The primary end point of the trial was the intima-media thickness measured by intravascular ultrasound at 1-year angiographic follow-up. Patients were screened to enroll 25 patients in each group based on a superiority design. Early postoperative clinical and angiographic outcomes were compared between the 2 groups.

**Results:**

The numbers of distal anastomoses per SV graft were 2.7 ± 1.1 and 2.6 ± 0.8 in the composite and aorta groups, respectively. There was no operative death, and postoperative complications in the 2 groups did not significantly differ. All patients underwent early postoperative graft angiography at a median of 1 (interquartile range, 1-1) day after operation. Early angiographic patency rates were 98.9% (93/94 anastomoses) and 100% (90/90 anastomoses) in the composite and aorta groups, respectively. There was no distal anastomosis showing competitive flow in the aorta group, whereas 3 anastomoses with competitive flow were found in the composite group. All 3 anastomoses with competitive flow were SV grafts that were anastomosed to the right coronary artery territory with moderate stenosis.

**Conclusions:**

Early clinical outcomes and angiographic graft patency rates were not significantly different with use of no-touch SV as a Y-composite graft or an aortocoronary graft.

## Visual Abstract

**Figure:**
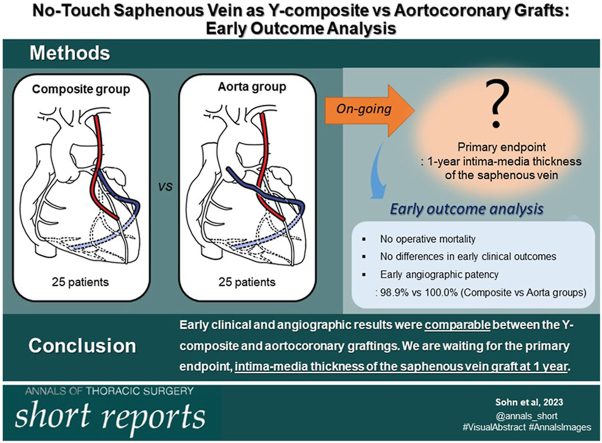

**In Short**

▪ The CONFIG trial was designed to compare the 1-year morphologic features of no-touch saphenous vein used as a Y-composite graft based on the in situ left internal thoracic artery with those of no-touch saphenous vein used as an aortocoronary graft by intravascular ultrasound.
▪ In this preliminary analysis, early postoperative clinical outcomes and angiographic patency rates were comparable between the groups.

Revascularization of the left anterior descending artery (LAD) with the left internal thoracic artery (LITA) has been recognized as the standard of care for coronary artery bypass grafting (CABG). However, the grafting strategy for non-LAD target vessels is still controversial. Despite the enthusiasm for multiple arterial grafting,^1^ the saphenous vein (SV) has been the most commonly used conduit for several decades.^2^ Recent advances, such as novel no-touch (NT) SV harvesting techniques and grafting strategies, have shown improved graft patency rates for the SV.^3^^,^^4^

Previous studies demonstrated favorable graft patency rates and negative remodeling of NT-SV when the SV was used as a Y-composite graft based on the in situ LITA.^4^^,^^5^ However, whether the favorable findings for NT-SV composite grafts were due to the effect of Y-composite grafting or the NT harvesting technique could not be conclusively determined because these 2 modifications were adopted at the same time during the study period; no study has directly compared the results of SV grafts harvested by the same technique but with different grafting strategies.

A randomized controlled trial entitled Morphologic Changes of the No-Touch Saphenous Vein as Y-Composite vs Aortocoronary Grafts in Coronary Artery Bypass Grafting (CONFIG; NCT04782492) was designed to compare the 1-year morphologic features of NT-SV used as a Y-composite graft based on the in situ LITA with those of NT-SV used as an aortocoronary graft. This study was conducted to introduce the CONFIG trial design and to evaluate early clinical and angiographic outcomes of the trial patients as a preliminary report.

## Material and Methods

### Study Design

**Figure 1:**
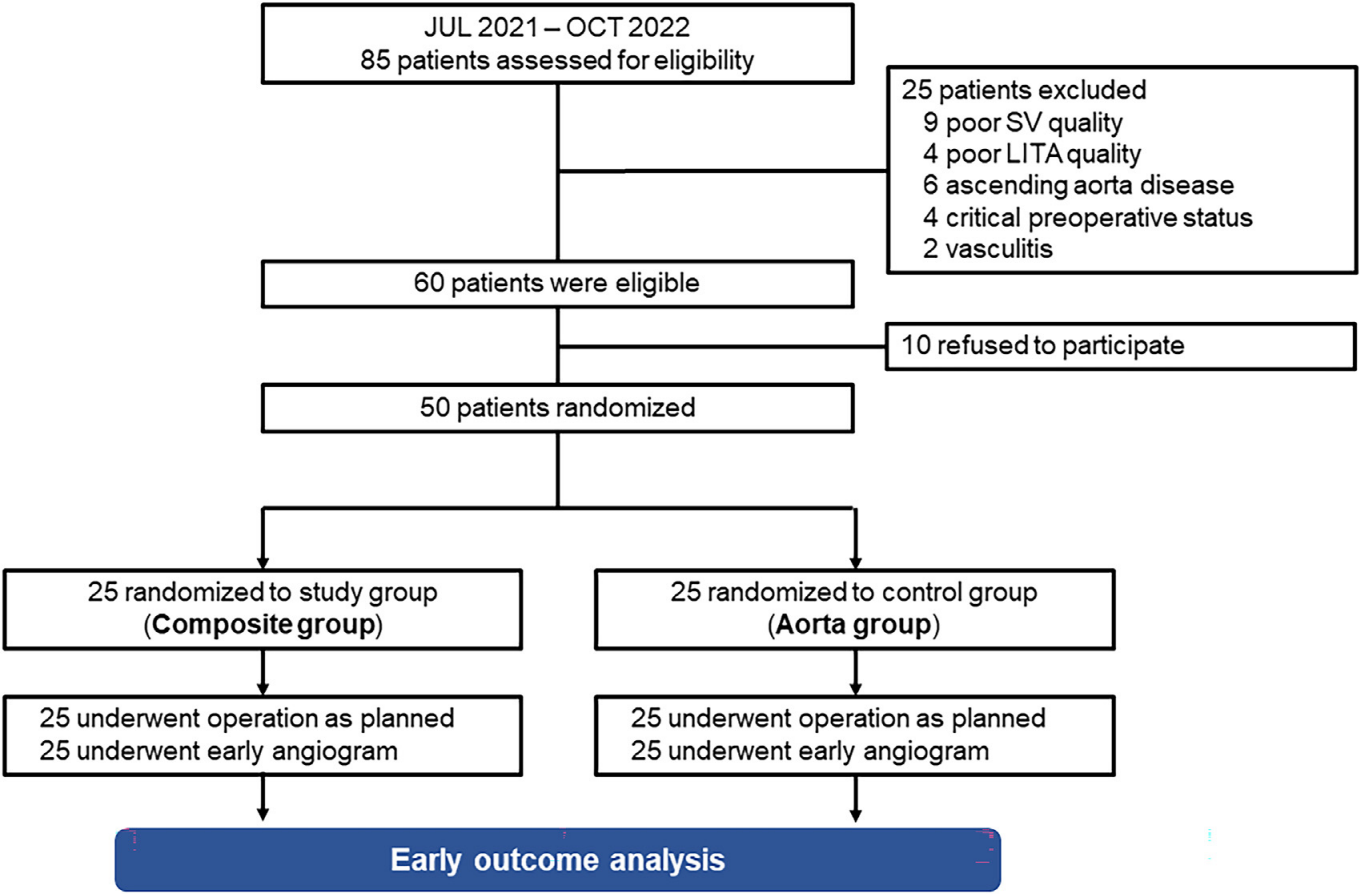
Flow diagram of the study population. (LITA, left internal thoracic artery; SV, saphenous vein.)

Patients older than 19 years who were scheduled to undergo primary isolated CABG for multivessel disease on a nonemergency basis and in whom the use of LITA and SV as bypass conduits was planned were assessed for eligibility for study enrollment (Supplemental Table 1 and Supplemental Study Protocol). Between July 2021 and October 2022, 85 patients were assessed for eligibility and 50 patients were enrolled. The study patients were randomly assigned to the Y-composite group (the composite group) or the aortocoronary group (the aorta group) in a 1:1 manner (Figure 1).

### Operative Strategies and Randomization Process

**Figure 2:**
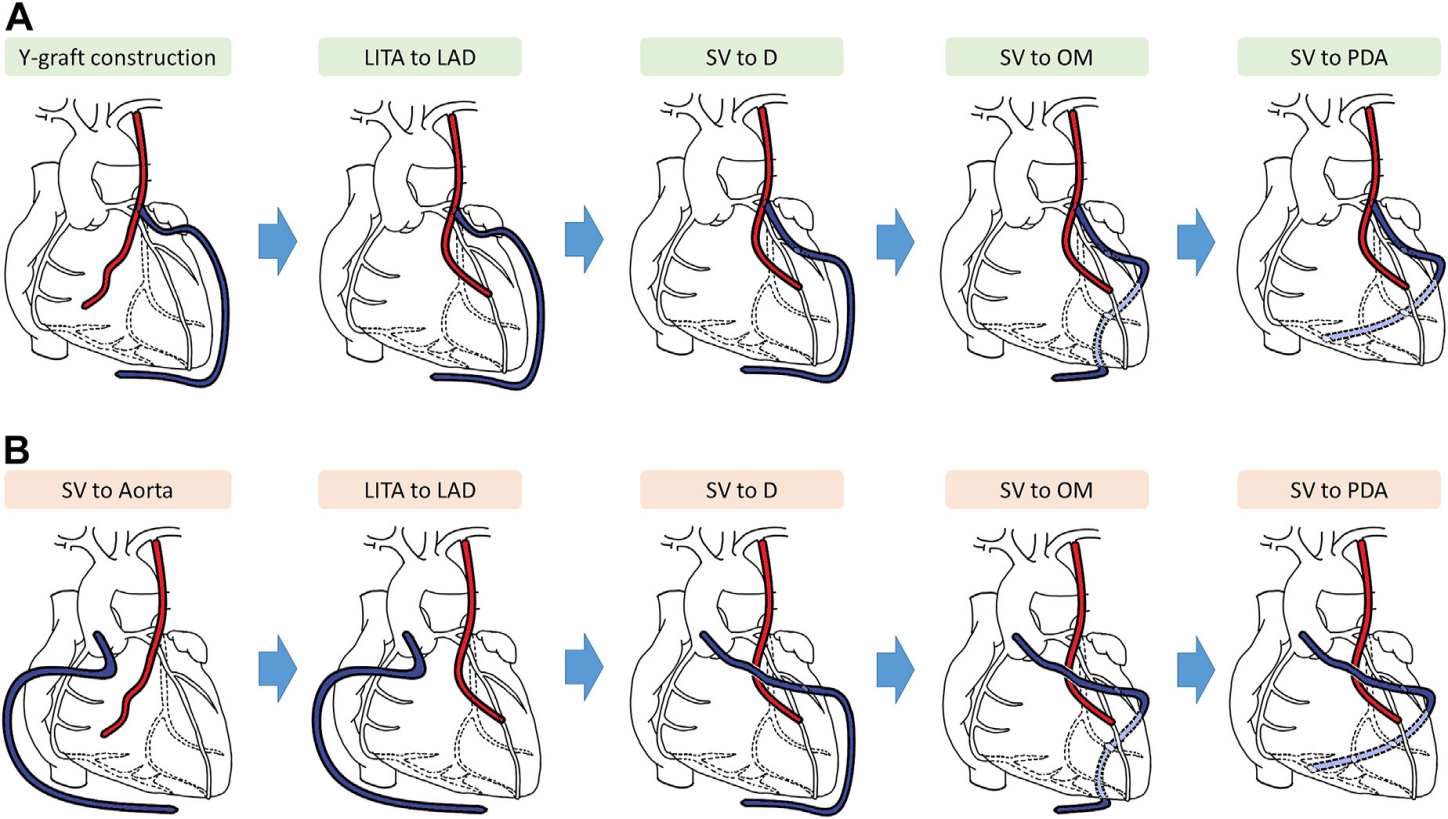
Procedural sequences for each grafting strategy. (A) Y-composite grafting strategy. (B) Aortocoronary grafting strategy. (D, diagonal branch; LAD, left anterior descending artery; LITA, left internal thoracic artery; OM, obtuse marginal; PDA, posterior descending artery; SV, saphenous vein.)

Off-pump CABG was the preferred grafting strategy during the study period. All diseased vessels with diameter ≥1.0 mm and stenosis degree of ≥50% in the LAD and left circumflex coronary artery territories and those with diameter ≥1.0 mm and stenosis degree of ≥70% in the right coronary artery (RCA) territory were considered the target vessels. The in situ LITA was harvested by the skeletonization technique. The SV was simultaneously harvested, preferentially from the lower leg, by an NT technique that retained perivascular soft tissue as previously described.^5^ Randomization was performed after the conduits had been harvested without injury and it had been confirmed by intraoperative findings that either of the grafting strategies was feasible. Web-based block randomization was performed with randomly determined block sizes of 4 and 6. After randomization, the procedures were performed according to the assigned grafting strategy. First, the NT-SV was connected to the LITA or ascending aorta as randomized, and anastomosis of the LITA to the LAD territory was performed. The SV was then anastomosed to the diagonal branch, to the vessels in the left circumflex coronary artery territory, and then to the vessels in the RCA territory as needed by a sequential anastomotic technique (Figure 2). All study patients underwent operation by a single surgeon who experienced >300 cases of off-pump CABG.

Forty-nine patients underwent off-pump CABG as planned. In the other patient, in the composite group, on-pump conversion was decided during off-pump LITA-to-LAD anastomosis because of hemodynamic instability, and the operation proceeded as an on-pump beating CABG.

### Evaluation of Early Outcomes

Early clinical outcomes, including operative mortality and postoperative complications, were evaluated. For the evaluation of graft patency, early postoperative graft angiography was performed at a median of 1 (interquartile range [IQR], 1-1) day in all the study patients.

### Statistical Analysis

The primary end point of the CONFIG trial was intima-media thickness (IMT) measured by intravascular ultrasound on 1-year angiography. Based on previous studies, the reference IMT was estimated as 0.31 ± 0.12 mm for the Y-composite configuration^5^ and 0.43 ± 0.09 mm for the aortocoronary configuration.^6^^,^^7^ The study was designed to have 90% power to detect a significant difference in IMT between the 2 groups with a 2-sided type I error of 5.0%. Based on this power calculation, 17 patients were needed in each group. Allowing a 30% dropout rate during the 1-year follow-up, recruitment of 50 patients (25 in each group) was determined to be necessary.

Statistical analyses were performed with SPSS software (version 25.0; IBM). Continuous variables are presented as the mean ± SD for normally distributed variables and as median with IQR for nonnormally distributed variables; categorical variables are presented as number and percentage of participants.

## Results

The median age was 65 (IQR, 61-69) years, and 20% (n = 10) were female (Table 1). The SV was harvested from the lower leg in 19 (76.0%) and 23 (92.0%) patients in the composite and aorta groups, respectively (Supplemental Table 2). The average numbers of distal anastomoses are presented in Supplemental Table 3.

**Table 1:** Preoperative Characteristics and Risk Factors of the Study Patients

Variable	Composite Group (n = 25)	Aorta Group (n = 25)	*P*
Age, y	64 (61-70)	65 (61-70)	.70
Sex, female	4 (16)	6 (24)	.73
EuroSCORE II	0.7 (0.6-2.0)	1.0 (0.7-1.3)	.41
Risk factors			
Smoking	15 (60)	13 (52)	.57
Body mass index >25.0 kg/m^2^	14 (56)	10 (40)	.26
Hypertension	17 (68)	15 (60)	.56
Diabetes mellitus	12 (48)	13 (52)	.78
Dyslipidemia	13 (52)	9 (36)	.25
History of stroke	0 (0)	3 (12)	.24
Chronic kidney disease	8 (32)	2 (8)	.07
Chronic obstructive pulmonary disease	0 (0)	1 (4)	>.99
Peripheral vascular disease	3 (12)	6 (24)	.46
Previous percutaneous coronary intervention	6 (24)	7 (28)	.75
Left ventricular ejection fraction <0.35	0 (0)	0 (0)	…
Acute coronary syndrome	13 (52)	11 (44)	.57
Three-vessel disease	17 (68)	18 (72)	.76
Left main disease	12 (48)	11 (44)	.78

Values are presented as number (percentage) or median (interquartile ranges).

There was no operative death. The occurrence rates of postoperative complications were not significantly different between the groups (Supplemental Table 4).

The early postoperative angiographic patency rates were 98.9% (93/94 anastomoses) and 100% (90/90 anastomoses) in the composite and aorta groups, respectively (Table 2). The 1 occluded anastomosis in the composite group was an anastomosis that was constructed with the SV to the obtuse marginal artery with a diameter <1 mm. In addition, there were 3 anastomoses with competitive flow in the composite group, whereas no anastomosis in the aorta group showed competition (Supplemental Figure). All 3 anastomoses with competitive flow in the composite group were SV grafts that were anastomosed to the RCA territory with moderate stenosis.

**Table 2:** Comparison of Early Postoperative Angiographic Patency Rates

Variables	Composite Group (n = 25)	Aorta Group (n = 25)
Overall	98.9 (93/94)	100 (90/90)
LITA	100 (26/26)	100 (26/26)
SV	98.5 (67/68)	100 (64/64)
Anastomosed to LAD territory	100 (21/21)	100 (21/21)
Anastomosed to LCX territory	96.3 (26/27)	100 (25/25)
Anastomosed to RCA territory	100 (20/20)^a^	100 (18/18)
Sequential anastomosis	100 (43/43)	100 (39/39)
Terminal anastomosis	96 (24/25)	100 (25/25)

Values are presented as % (n/N).

LAD, left anterior descending; LCX, left circumflex; LITA, left internal thoracic artery; RCA, right coronary artery; SV, saphenous vein.

a Three of 20 anastomoses showed competitive flow.

## Comment

This study demonstrated 2 main findings. First, early clinical outcomes and angiographic patency rates did not differ significantly between the composite and aorta groups. Second, several competitive flows were observed in the composite group when NT-SV was anastomosed to the vessels with moderate stenosis in the RCA territory.

The SV, as a conduit for CABG, demonstrated improved patency when it was harvested with the NT technique and used as an aortocoronary graft.^3^ Backgrounds for improved patency of the NT-SV include saving the vasa vasorum that protects the SV from transmural ischemic damage, buffering against pulsatile stress of the arterial flow that reduces neointimal and medial thickening, preserving perivascular tissues in which adipocytes interact with vascular smooth muscle cells and modulate vascular tone,^8^ and releasing nitric oxide that plays a crucial role in suppressing atherosclerosis.^9^

In addition to advances in SV harvesting technique, SV grafting strategy using Y-composite grafting based on the in situ LITA was reported to have resulted in excellent graft patency comparable to that of arterial grafts.^4^ The theoretical advantages of SV composite grafting based on the in situ LITA have been suggested to be exposure to less circulatory stress than with SV aortocoronary grafts and the production of endothelial-protective substances, such as nitric oxide, by the LITA that flow down to the composite SV graft. Other studies also demonstrated that SV composite grafts underwent favorable negative remodeling during the first year after CABG without any abnormal growth of the intima^5^ and with improved endothelial shear stress.^10^ However, it is unclear whether these findings are specifically associated with the composite grafting strategy because NT-SV harvesting was also used in all of these studies.

Competitive flow was observed in several patients from the composite group, which is 1 of the concerns in performing CABG by a composite grafting strategy based on the in situ LITA. Competitive flow can be observed at either the LITA-to-LAD anastomosis or other anastomoses constructed with the second conduit if 1 arm has high-flow demand from the LITA and the other has low-flow demand because of a moderate degree of stenosis of the native vessels.

The CONFIG trial was conducted to compare 1-year results after CABG using the NT-SV as a composite graft based on the LITA with those after CABG using the NT-SV as an aortocoronary graft. The final report could clarify whether the composite grafting strategy provides any additional benefits for outcomes after CABG using the NT-SV.

The study has several limitations that must be recognized. First, small sample size would be a limitation of this randomized trial. Second, this study presented only early outcome analysis of a planned randomized trial. The trial has completed patient enrollment, and patients are now being followed up through 1-year intravascular ultrasound evaluations. After reaching the primary end point, the patients will be followed up 5 years and beyond.

## Supplementary Data

Supplemental Tables 1-4 and Supplemental Figure 1

Supplemental Study Protocol

## References

[1] Chikwe J., Sun E., Hannan E.L. (2019). Outcomes of second arterial conduits in patients undergoing multivessel coronary artery bypass graft surgery. J Am Coll Cardiol.

[2] Schwann T.A., Tatoulis J., Puskas J. (2017). Worldwide trends in multi-arterial coronary artery bypass grafting surgery 2004-2014: a tale of 2 continents. Semin Thorac Cardiovasc Surg.

[3] Souza D.S., Johansson B., Bojo L. (2006). Harvesting the saphenous vein with surrounding tissue for CABG provides long-term graft patency comparable to the left internal thoracic artery: results of a randomized longitudinal trial. J Thorac Cardiovasc Surg.

[4] Kim K.B., Hwang H.Y., Hahn S., Kim J.S., Oh S.J. (2014). A randomized comparison of the Saphenous Vein Versus Right Internal Thoracic Artery as a Y-Composite Graft (SAVE RITA) trial: one-year angiographic results and mid-term clinical outcomes. J Thorac Cardiovasc Surg.

[5] Hwang H.Y., Koo B.K., Oh S.J., Kim K.B. (2015). Morphologic changes of the saphenous vein Y-composite graft based on the left internal thoracic artery: 1-year intravascular ultrasound study. J Thorac Cardiovasc Surg.

[6] Johansson B.L., Souza D.S., Bodin L. (2010). Slower progression of atherosclerosis in vein grafts harvested with 'no touch' technique compared with conventional harvesting technique in coronary artery bypass grafting: an angiographic and intravascular ultrasound study. Eur J Cardiothorac Surg.

[7] Taggart D.P., Ben Gal Y., Lees B. (2015). A randomized trial of external stenting for saphenous vein grafts in coronary artery bypass grafting. Ann Thorac Surg.

[8] Dashwood M.R., Savage K., Tsui J.C. (2009). Retaining perivascular tissue of human saphenous vein grafts protects against surgical and distension-induced damage and preserves endothelial nitric oxide synthase and nitric oxide synthase activity. J Thorac Cardiovasc Surg.

[9] Saito T., Kurazumi H., Suzuki R. (2022). Perivascular adipose tissue is a major source of nitric oxide in saphenous vein grafts harvested via the no-touch technique. J Am Heart Assoc.

[10] Hwang H.Y., Koo B.K., Yeom S.Y., Kim T.K., Kim K.B. (2018). Endothelial shear stress of the saphenous vein composite graft based on the internal thoracic artery. Ann Thorac Surg.

