# Supplementary material for: No-Touch Saphenous Vein as Y-Composite vs Aortocoronary Graft: Early Outcome Analysis
Source: Ann Thorac Surg Short Rep. 2023 Oct 4;2(1):30–4. doi: 10.1016/j.atssr.2023.09.015 (PMC11708128; doi:10.1016/j.atssr.2023.09.015)

**Supplemental material**

SUPPLEMENTAL TABLE 1. Randomization criteria of the trial.

|  | Criteria |
| --- | --- |
| Inclusion | (1) Patients who are scheduled to undergo primary isolated CABG for a multivessel disease  (2) Patients over 19 years of age  (3) Patients who are planned to use of LITA and SV as bypass conduits  (4) Patients who or whose legal representative fill out a written consent form before the start of the clinical trial and patients who can comply with the clinical trial requirements |
| Exclusion | (1) Patients with concomitant cardiac procedures including valve or aorta surgery  (2) Patients in whom it is not feasible to use the LITA or the SV as a bypass conduit due to intrinsic problems with the vessels  (3) Patients with severely atherosclerotic or calcified ascending aorta that precludes the use of aortocoronary anastomosis of the SV  (4) Patients who have vasculitis  (5) Patients with severe comorbidities that may limit the possibility of 1-year angiographic follow-up  (6) Patients who refused study enrollment |

SUPPLEMENTAL TABLE 2. Location of the harvested saphenous vein.

|  | Total  (n = 50) | Composite group  (n = 25) | Aorta group  (n = 25) | *P* |
| --- | --- | --- | --- | --- |
| Lower leg, n (%) | 42 (84.0%) | 19 (76.0%) | 23 (92.0%) | .25 |
| Left | 31 (62.0%) | 13 (52.0%) | 18 (76.0%) | .15 |
| Right | 11 (22.0%) | 6 (24.0%) | 5 (20.0%) | >.99 |
| Upper leg, n (%) | 8 (16.0%) | 6 (24.0%) | 2 (8.0%) | .25 |
| Left | 5 (10.0%) | 5 (20.0%) | 0 (0.0%) | .05 |
| Right | 3 (6.0%) | 1 (4.0%) | 2 (8.0%) | >.99 |

SUPPLEMENTAL TABLE 3. Comparison of the numbers of distal anastomoses between the 2 groups.

| Variables | Total  (n = 50) | Composite group  (n = 25) | Aorta group  (n = 25) | *P* |
| --- | --- | --- | --- | --- |
| Per patient | 3.7 ± 1.0 | 3.8 ± 1.2 | 3.6 ± 0.8 | .58 |
| Per LITA | 1.0 ± 0.2 | 1.0 ± 0.2 | 1.0 ± 0.2 | >.99 |
| Per SV | 2.6 ± 1.0 | 2.7 ± 1.1 | 2.6 ± 0.8 | .57 |
| Anastomosed to LAD territory | 0.8 ± 0.5 | 0.8 ± 0.6 | 0.8 ± 0.4 | >.99 |
| Anastomosed to LCX territory | 1.0 ± 0.6 | 1.1 ± 0.7 | 1.0 ± 0.5 | .65 |
| Anastomosed to RCA territory | 0.8 ± 0.6 | 0.8 ± 0.6 | 0.7 ± 0.6 | .66 |

Values are presented as mean ± standard deviation

LAD, left anterior descending; LCX, left circumflex; LITA, left internal thoracic artery; RCA, right coronary artery; SV, saphenous vein.

SUPPLEMENTAL TABLE 4.Comparison of early clinical outcomes between the 2 groups.

| Variables | Total  (n =50) | Composite group  (n = 25) | Aorta group  (n = 25) | *P* |
| --- | --- | --- | --- | --- |
| Operative mortality, n (%) | 0 (0.0) | 0 (0.0) | 0 (0.0) | - |
| Postoperative complications, n (%) |  |  |  |  |
| Postoperative atrial fibrillation | 11 (22.0) | 4 (16.0) | 7 (28.0) | .50 |
| Acute kidney injury | 1 (2.0) | 0 (0.0) | 1 (4.0) | >.99 |
| Respiratory complications | 1 (2.0) | 1 (4.0) | 0 (0.0) | >.99 |
| Delirium | 1 2.0) | 1 (4.0) | 0 (0.0) | >.99 |
| Bleeding reoperation | 0 (0.0) | 0 (0.0) | 0 (0.0) | - |
| Stroke | 0 (0.0) | 0 (0.0) | 0 (0.0) | - |
| Mediastinitis | 0 (0.0) | 0 (0.0) | 0 (0.0) | - |

SUPPLEMENTAL FIGURE 1. Early postoperative angiograms demonstrating competitive flow of the SV grafts. (A) Graft angiogram showed patent LITA-to-LAD anastomosis and patent anastomosis of the SV to diagonal and obtuse marginal branches. However, the posterior descending artery was not visualized on this graft angiogram due to competitive flow (*white arrow*). (B) Native RCA angiogram showed patent anastomosis of the SV graft to the posterior descending artery which was visualized with retrograde competitive flow (*black arrow*). LAD, left anterior descending artery; LITA, left internal thoracic artery; RCA, right coronary artery; SV, saphenous vein.


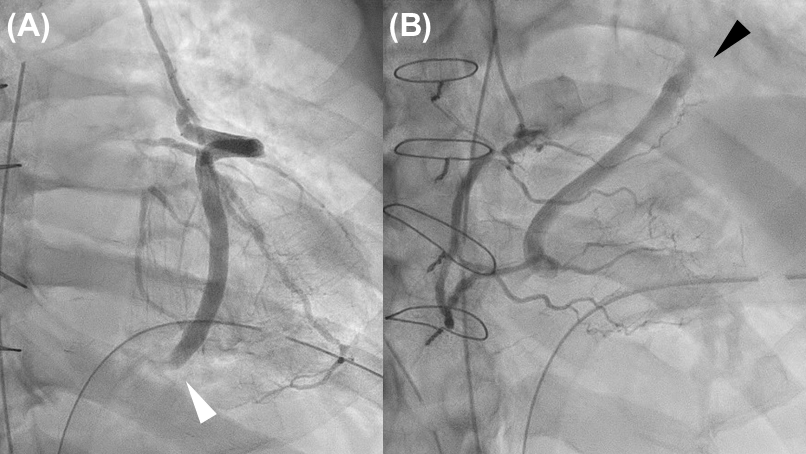

Supplement: Supplemental Tables 1-4 and Supplemental Figure 1 [file mmc1.docx]
